# Fertility preservation and protection: young women’s decision-making about contraceptive use in Zimbabwe

**DOI:** 10.1080/13691058.2023.2258175

**Published:** 2023-09-20

**Authors:** Constancia V. Mavodza, Constance R.S. Mackworth-Young, Rangarirayi Nyamwanza, Portia Nzombe, Ethel Dauya, Chido Dziva Chikwari, Mandikudza Tembo, Rashida A. Ferrand, Sarah Bernays

**Affiliations:** a The Health Research Unit, https://ror.org/0130vhy65" Biomedical Research and Training Institute, Harare, Zimbabwe; b Department of Public Health, Environments and Society, Faculty of Public Health and Policy, London School of Hygiene and Tropical Medicine, London, UK; c Department of Global Health and Development, Faculty of Public Health and Policy, London School of Hygiene and Tropical Medicine, London, UK; d Department of Infectious Diseases Epidemiology, Faculty of Epidemiology and Population Health, London School of Hygiene and Tropical Medicine, London, UK; e Clinical Research Department, Faculty of Infectious and Tropical Diseases, London School of Hygiene and Tropical Medicine, London, UK; f School of Public Health, University of Sydney, Sydney, Australia

**Keywords:** Contraception, fertility, decision-making, young women, Zimbabwe

## Abstract

The study explored social and health system influences on young women’s decision-making about family planning in a community setting with low uptake. Seventy-two semi-structured interviews were conducted between April 2020 and November 2021, with both young women accessing, and healthcare workers providing, a community-based integrated package of HIV and sexual and reproductive health services (CHIEDZA) in Zimbabwe. Data were thematically analysed. Although long-acting contraception was freely available as part of the CHIEDZA initiative, uptake was low. Young women’s contraception choices were influenced by a desired reproductive sequence, which reflected prevailing social norms and was conveyed by peers and female relatives. Nulliparous young women preferred short-term contraception and avoided hormonal contraceptives prepartum to ‘preserve’ their fertility. Once fertility had been confirmed within marriage through the birth of a child, hormonal contraceptive use became socially permissible. Healthcare workers, cognisant of community discourse, sensitively proposed alternative approaches. Increasing the availability of correct and adequate information and commodities is critical to improving the uptake of contraceptives for young women, but it is insufficient alone. Recognising and responding to local contextual understandings which frame considerations of appropriateness is paramount. Successful implementation of family planning interventions requires engaging with social norms and the influential groups that perpetuate them.

## Introduction

Sub-Saharan Africa has the highest rates of teenage pregnancies in the world (Ahinkorah et al. 2021). Within the region, approximately 35% of the pregnancies in girls below 18 are unintended, indicating an unmet need for family planning (Chae et al. 2017; Izugbara and Egesa 2014). Where young women do take up family planning options, they often use short-term methods such as oral contraceptives and condoms that have high failure and discontinuation rates (Radovich et al. 2018; Chandra-Mouli et al. 2014; Willan et al. 2020), compared to long-term methods such as implants and intra-uterine devices.

Substantial investment in modern contraceptive access has led to Zimbabwe having one of the highest modern contraceptive prevalence rates for all women of reproductive age. The modern contraceptive prevalence rate is 65% (ZIMSTAT and ICF International 2016), compared to an average of 29% for the rest of the region (Ahinkorah et al. 2021). However, a high unmet family planning need for young unmarried women persists at 37% for 15–19 year olds and 17% for 20–24 years compared to 12.6% overall for women of reproductive age (15–49 years) in the country (ZIMSTAT and UNICEF 2019; MoHCC 2016). This suggests that specific factors impede access, which disproportionately affect young women.

Demand side factors that can prevent young women from using family planning include socio-cultural expectations, denial of young women’s sexuality, and stigma concerning contraceptive use (Chandra-Mouli et al. 2014; WHO 2017). Many of these demand side factors are gendered (McClendon et al. 2018) as they primarily affect young women and not their male counterparts. Supply side factors include discrimination by health providers, confidentiality concerns, and commodity availability challenges, which can result in limited service provision for young women as well as a reduced willingness to engage with family planning services (WHO 2017; Denno, Hoopes, and Chandra-Mouli 2015).

Sexual and reproductive health (SRH) interventions which seek to address these challenges have demonstrated limited sustained impact on reducing unintended pregnancy outcomes among young women (Phillips and Mbizvo 2016; Smith 2020). This indicates the need to further explore ‘access’ beyond supply and availability to better understand the influences that may shape young women’s decision-making and uptake (de Vargas Nunes Coll et al. 2019; Mutumba, Wekesa, and Stephenson 2018; Smith 2020), and to examine how this may differ by contraception method.

Many health service utilisation theories consider the point of contact with the formal health system as being the most important for understanding access to and use of these services (Andersen 2008; Ricketts and Goldsmith 2005). However, experiences and behaviours outside of the health care service delivery system are significant contributors to young people’s family planning decision-making and health seeking behaviours. For example, as part of socio-cultural norms, in some contexts, unequal gender power relations are significant in reproductive decisions, including when to use contraceptives, or what type to use (Mosha, Ruben, and Kakoko 2013; Schuler, Rottach, and Mukiri 2011). These, in turn, shape willingness to engage with the health system (Starrs et al. 2018; Stackpool-Moore et al. 2017).

The World Health Organization (WHO) has made a public health case for making a wide range of family planning options available to young women, including long-acting contraception which can provide discreet and ongoing protection (WHO 2011, 2017). Young women in sub-Saharan Africa tend to take up short-acting contraceptives (Radovich et al. 2018; Chandra-Mouli et al. 2014), but limited attention has been paid to articulating whether they do this in the presence or absence of mixed methods (short and long-acting) contraceptives.

This paper examines whether, and how, young women consider themselves suitable candidates to take up hormonal contraception, if it is freely available to them. We aim to provide evidence that can inform the approaches to framing family planning options within interventions, so that they can better align with young women’s needs, to improve health outcomes.

## Materials and methods

### Study design

This qualitative study was conducted as part of the nested process evaluation of a community-based integrated HIV and sexual reproductive health service. The service included offering family planning, for young people aged 16–24 years, and was evaluated through a cluster-randomised trial in Zimbabwe (CHIEDZA—trial registration number NCT03719521).

### Study setting

The CHIEDZA trial was conducted in three provinces in Zimbabwe: Harare, Bulawayo and Mashonaland East between April 2019 and March 2022. Details of the CHIEDZA intervention are published elsewhere (Dziva Chikwari 2022; Mackworth-Young et al. 2022). Evidence indicates that CHIEDZA was generally perceived to be available and accessible to young people (Mavodza et al. 2021; Tembo et al. 2022).

### Family planning in CHIEDZA

Family planning services within CHIEDZA included the provision of information, counselling, and contraceptive commodities by youth-friendly and family planning trained providers. Oral contraceptives (short-term methods) and Depo-Provera injectables (medium-term method) were supplied by the project nurses. Between April 2019 and October 2020, long-acting reversible contraceptives (LARCs), specifically implants and Intra-uterine Devices (IUDs), were provided through offsite referrals to a partner non-governmental organisation, Population Services Zimbabwe, which operates in public sector clinics and its own centres. From October 2020, LARCs were provided onsite at the trial’s community centres by this organisation. Ideally, LARC provision should have been present every time that CHIEDZA was open (same day each week), but this was not always feasible. In the non-governmental organisation’s absence, the referral pathway to other health facilities was used.

### Data collection

Data were collected in five phases, three with service providers (phases 1, 3 and 5) and two with female service attendees (phases 2 and 4), referred to as clients. Data were collected across all provinces between April 2020 and November 2021. Each phase of work informed subsequent phases. The data collection phases were also informed by the routine (quantitative) family planning services uptake data. A total of seventy-two semi-structured interviews were conducted (Table 1).

Due to COVID-19 lockdown mobility restrictions around the time of the study, interviews were conducted both in-person and telephonically, depending on lockdown restrictions at the time (Table 1). Interviews were conducted by qualified qualitative researchers, who were not directly involved in service provision (CVM, RN and PN). Each interview took between 15–90 min, and on average lasted approximately 45 min. Written informed consent was provided prior to each interview.

### Provider interviews

A total of 42 interviews were conducted with the same group of service providers in phase 1 (*n* = 16), phase 3 (*n* = 15), and phase 5 (*n* = 11) (Table 1). Only 11 providers were interviewed in phase 5 as data saturation had been reached. In phase 1, the interviews broadly explored the family planning issues that arose in their discussions with clients. In phase 3, the interviews explored whether, how and why these issues and concerns persisted, as well the providers’ perceptions of these concerns. In phase 5, the interviews explored providers’ experiences of addressing these concerns during the implementation of the project.

### Client interviews

CHIEDZA client interviews were conducted in phase 2 (*n* = 13) and phase 4 (*n* = 15) (Table 1). The phase 2 interviews sought to generate a broad understanding of young people’s decision-making about family planning. In phase 2, female youth community mobilisers (*n* = 13) were interviewed because of their distinct perspectives in being clients, and their roles within the project. As youth mobilisers, they sensitised the trial intervention communities and mobilised their peers to access the intervention. This positioned them well to understand their own needs as young people, how young people responded to the trial, as well as the ideas circulating within their communities.

The phase 4 interviews (*n* = 15) consisted of a purposive sample of participants who were female, not having participated in phase 2, using contraceptive methods and in a diverse range of relationship situations (Table 2). Participants who were using a variety of different contraceptive methods were selected to understand perceptions, beliefs, and experiences by contraceptive method. The participants were selected from three clusters (one per province) where LARC provision by the partner organisation had been most consistent, to enable sufficient recruitment of young women who took up LARCs.

In phase 4, seven interviews were conducted using a semi-structured topic guide. The remaining eight interviews were unstructured and conducted without a topic guide, to elicit young women’s narratives about SRH. In this unstructured style, the researchers (RN and CVM) used a conversational format to talk about fertility, sex, pregnancy, and contraceptive use with participants. The semi-structured interviews conducted in this phase, alongside the prior rounds of data collection, were flexible in the topics discussed and responsive to additional issues raised by participants, but they predominantly covered subjects the research team had determined as priority areas. We adopted an unstructured approach with eight participants to enable them to have greater control over the topics discussed. Despite the slight variation in approaches, topics covered were similar between the two approaches.

### Data analysis

All interviews were conducted in the participant’s preferred language (English, Shona, or Ndebele) and audio recorded. Each interview was transcribed directly into English by bilingual researchers. To maintain confidentiality, de-identification was undertaken during transcription.

The analysis was guided by the principles of interpretive thematic analysis (Braun and Clarke 2006). CVM read all transcripts to familiarise with the data. Data were coded, and the initial inductive codes were developed, including ‘contraceptive use postpartum’ and ‘contraceptive use within marriage’. Through this initial coding process, social influences on young women were identified as important issues for further attention to explore their effect on decision-making and why. Inductive codes such as ‘fertility concerns’, ‘information sources’ and ‘hormonal contraceptive side-effects’ from the whole data set were compiled in data summary notes using Microsoft Word. Analytical memos were drafted to explore connections between codes and further develop emerging ideas, and to highlight and arrange significant themes (Birks, Chapman, and Francis 2008). To advance the analysis, coded excerpts from the transcripts and data summaries were grouped together under the identified themes presented in this paper. The analytical processes were iterative and involved collaborative discussion between CVM and SB.

Anonymised quotes from the providers are described with only the interview number, province and data collection phase details to protect anonymity. Quotes from clients are described by interview number, type of contraceptive used, marital status, and data collection phase. In Zimbabwe, *Secure* is the brand name of the progesterone-only contraceptive pill (PoP), *Control* is the brand name for combined oral contraceptive (COC) pills, *Jadelle* is a brand name for an implant and Depo is the shortened name referring to Depo Provera injectable. If a participant descriptor has two or more methods, it indicates that the individual concerned had switched methods.

### Ethics

Ethical clearance for the project was obtained from the Medical Research Council of Zimbabwe (MRC/A/2266), the London School of Hygiene and Tropical Medicine (14652), and the Biomedical Research and Training Institute in Zimbabwe (AP144/2018). Written informed consent for participation was obtained from all participants.

## Results

The intervention’s routine services data showed a low uptake of family planning services. Of the total 27,275 women who ever accessed services, only 38.7% (10,721) took up family planning services; and less than 4% of these took up long-acting contraception. The results presented in this section identify factors beyond the health service availability and acceptability that may influence uptake and engagement.

Table 2 outlines the key characteristics of this study’s participants. Many young women described themselves as married, although their situations may not have met the local, or legal threshold of the definition of marriage. We intentionally used the descriptor provided by the young women themselves to determine their marital status.

All participants in phase 4 had children and many described their reproductive journeys retrospectively. Three of the participants in phase 2 were sexually active, nulliparous (of reproductive age, and has never given birth to a child) and considered themselves unmarried. The diversity of the circumstances of the young women in the study provided insights into both present and past lived experiences of contraceptive decision-making.

### Proving fertility: an embodiment of womanhood

The socially and morally acceptable sequence of sexual and fertility events for young women was as follows: no sex before marriage, marriage, no hormonal contraceptives before proving fertility, and then going on to have a baby once married. Among the clients interviewed, great importance was placed on young women proving their fertility through childbearing to establish themselves as embodying a socially acceptable womanhood, prior to using contraceptives. This socially anticipated sequence of events was very influential in shaping young women’s decision-making about family planning options.

You must have a baby first then think of using any family planning you wish to use. (IDI04, COC-Depo, married, Phase 4)

Most of the participants perceived that a hormonal contraceptive was best suited for married women and only once they had had at least one child.

I know that when you get married you don’t start by using family planning. You must have a baby first then think of using any family planning you wish to use. When you want to have your second child that’s when you start using it so that there is the spacing between your first and your second child. (IDI04, COC-Depo, married, Phase 2)

### Protecting fertility

A widely held assumption was that hormonal contraception could disrupt a young woman’s future fertility. This perceived side effect significantly shaped why such contraception was thought to be avoided by nulliparous women. Hormonal contraception was seen as a direct threat to young women’s reproductive intentions, and in turn to their projected attainment of womanhood.

I wouldn’t want to use the contraceptives before getting married because maybe it would lower the chances of me getting pregnant. I think since the uterus wouldn’t have carried a child before, it might become complicated for an unmarried woman to start using the contraceptives. (IDI02, COC, married, Phase 4)

Both providers and client participants cited side effects as the primary reason for low uptake of hormonal contraception among young unmarried women and their preference for condoms, or in many cases deliberately avoiding hormonal contraception altogether.

I feared to tarnish my reproductive health before I have started bearing children, hence the choice of condoms. (IDI10, condoms only, in a relationship, Phase 2)

I was not using anything [before having a child] … I heard that one should never use family planning, especially if they do not have a child. I was told that it makes one infertile and they may face challenges when they now want to have a baby and so I did not want that to happen to me. (IDI15, Jadelle, in a relationship, Phase 4)

### Performing the moral trajectory of womanhood

The expectation to prove one’s fertility was layered onto moral codes concerning sexual debut and marriage. The social ideal was that young women were expected to have their sexual debut within a marital situation. At that point, the expectation shifted towards proving fertility by having a baby soon after marriage.

However, the trajectory of being married before becoming sexually active was not consistently adhered to. Participants’ accounts illuminate the malleability of both what might constitute marriage and what could become an ‘approved’ reproductive trajectory in attaining the status of womanhood. Some young women perceived that being sexually active with a consistent partner, was a proxy indicator for marriage. For example, for the participant quoted below it was the event of pregnancy which conferred her status shift into being married and only post-partum did she then take up hormonal contraception:

I got married as a result of the pregnancy. When we were boyfriend and girlfriend, we used to sleep together. When I later found out that I was pregnant. I had to go and stay with my boyfriend. It has been two years now so I can safely say he is now my husband. (IDI14, Jadelle, married, Phase 4)

Participants’ accounts emphasised that marital status could be conferred retrospectively and informally. Despite the negotiability of whether a couple was seen as married, becoming a mother through bearing a child was publicly visible and a clearly definable state. Even if a young woman’s marital status was somewhat ambiguous at the time of childbirth, bearing a child carried social value and conferred an elevated status on young women through their performative attainment of a critical element of womanhood. Some young women had been, or were sexually active prior to marriage, and therefore had already deviated from the socially approved sequence. However, refusing to engage in family planning methods or services until after having had a baby (usually) within marriage, was an opportunity to demonstrate a compliance to social expectations.

In phase 4 of data collection, 13 of the 15 women reported only ever using hormonal contraceptives post-partum (Table 2). For these 13 women, intervening to control fertility through hormonal contraception, after having demonstrated fertility, carried little social risk. Instead, their transition in status warranted contraceptive use to become a socially permissible option that enabled responsible planning for subsequent pregnancies. In phase 2, all the nine young women who were using only condoms or no contraception at all, did not yet have any children.

So those who take family planning are married people who have newly born babies so that they don’t have babies after every year or less. (IDI11, no contraception, single, Phase 2).

### Acceptability thresholds for different contraceptive methods

Although prioritising the perceived preservation of fertility was a general trend within the dataset, there was some variation in the degree to which it shaped method choices. Even once LARCs had become a socially permissible option for participants who already had children, some were still very wary of their perceived fertility-damaging side effects. This influenced the choices they made and limited the appeal of the longer-acting contraceptive options.

There were a few widely circulating misconceptions about LARCs which undermined young women’s confidence in their suitability as an option for them. One perception was that the fertility threat increased proportionate to the length of time a woman was using hormonal contraception. Specifically, participants considered that time on contraception was equivalent to the time it would take to conceive once they had stopped taking contraception. For example, one young mother chose not to take *Jadelle* because of her concerns that it might provoke a repetition of her previous conception challenges once she was ready to have another baby:

I thought for me to use *Jadelle* it may take about 5 more years for me to conceive. I decided not to use it since I took about 1 year and 9 months without a child. So, *Jadelle* was a no for me. I decided to go for the Depo and thought to myself that if 3 months lapses and I decide that I want a child I will not go back to the clinic for another shot; simple as that. (IDI10, Depo, married, Phase 4)

Other women also selected to use Depo, which acts for only three months. Depo was a convenient medium-term method that allowed them to ‘check’ their fertility by stopping periodically and allowing their menstrual cycle to return. They preferred this option because it provided reassurance about their ongoing reproductive potential.

I prefer Depo because of its short effective period. After three months, I can go back to my menses cycle. unlike *Jadelle* which is effective for 3 years, thus a long time for someone to be missing her menses… I like the fact that each time I come for my jab I am tested for pregnancy. (IDI13, Depo, in a relationship, Phase 4)

However, service providers highlighted that there were community rumours about the fertility-damaging side effects of Depo as well, which further discouraged uptake of this medium-term option.

Information went viral that if you have only one child you are not supposed to use Depo; if you decide to have another baby you will face some conception problems. So, it was wrong information being given, which made Depo less popular. (Harare Provider, IDI02, Phase 5)

### Diverging from the norm

There were exceptions (*n* = 3) to the dominant pattern of prioritising the desire to prove one’s fertility over protecting against conception when deciding which contraceptive option to use (*n* = 25). The three exceptions who were amongst the eldest within the sample, were more persuaded by public health rationales than prevailing social expectations within their communities. These women considered contraception to be appropriate for sexually active individuals, independent of marital or fertility status, who wanted to prevent unintended pregnancies.

For someone who is sexually active but not married, I think they should get a long-term family planning so that they don’t risk having unwanted pregnancies when the time isn’t ripe yet for them to have one. I would recommend that person to use loop or *Jadelle* which are long term methods because that will be the safest thing to do. (IDI12, PoP-Jadelle-Jadelle removal, married, Phase 4)

However, the divergent participants’ preference for LARCs was not only because they considered them protective against unintended pregnancies. LARCs also enabled them to be discreet, which protected the confidentiality of their contraceptive choices. This made long-term contraceptives preferable compared to the contraceptive pill which, in needing to be orally taken each day, risked them being ‘found out’. If their contraceptive choices were known about, this might draw unwelcome attention to their deviation from social norms and provoke social sanctions. These women recognised that if they needed to take the pill in secret it would potentially disrupt their ability to adhere, and undermine its effectiveness:

People will start asking you why you are taking family planning pills, yet you don’t have a husband. Others will also make me hide them to the extent that I will forget where I hid them and eventually misplace or lose them. (IDI08, COC, in a relationship, Phase 2)

### Sources of information and influence

Young women’s decisions about contraception were shaped by the social meanings attributed to the contraceptive options rather than clinical evidence. These social meanings were conveyed by trusted adults, as well as peers, within their community, who drew on their lived experience or the reported learning of others. As each story and advice that young women heard from those in their community tended to reinforce each other, the consistency of the accounts and advice they heard reinforced its status as ‘truth’. As such, these influential individuals mediated the perceived choices available to young women.

It’s not only my friends who said that [taking hormonal contraception will make it difficult to conceive], also people from my community said the same thing. Since a lot of people are saying that it could mean it’s true. (IDI14, Jadelle, Married, Phase 4)

### Role of providers in shaping decisions

Young women also trusted health providers who provided them with family planning information. Before they came to CHIEDZA, some young women had been told about infertility being a contraceptive side effect by health providers at their local clinics. One young woman was still in school, and not yet ready to have a baby. She got pregnant because she was not using any hormonal contraceptives, and she said it was because:

I was given these teachings at the local clinic. We were told it’s not advisable to use family planning before you have a child. The providers at the clinic just advised us to use condoms. I used to track my days and not have sex when I was ovulating. I guess that I was not an expert on that which is why I fell pregnant because my partner never loved using condoms. (IDI13, Depo, in a relationship, Phase 4).

When young women then attended CHIEDZA, much of the information that they were given about family planning options contradicted what they had come to believe through community discourse or from other health care workers. CHIEDZA providers acknowledged that the information that they were providing, which suggested that all family planning options were appropriate for sexually active young women, was in tension with local understanding about who family planning should be for. They attempted to correct the instructions that young women had previously been given by providing accurate information on all the contraceptive methods so that young women could make an informed decision on their method of choice.

Information that people in the community have is false. They say that implants are irreversible and if you decide to have a baby you will face many problems. So that’s another issue on long term methods, that’s why these young people didn’t want to use them… But it also takes time for one to understand all the information, and with time the clients would come back and switch the methods from pills to implants. (Harare Provider, IDI02, Phase 5)

Some providers acknowledged that they were also influenced by the social expectations about what was appropriate for a young woman regarding use of family planning methods.

From my beliefs I think young adolescents do not need those services [family planning]. At that age it’s time to discover yourself and focus more on growing yourself as an individual instead of intimacy. But I do not let my beliefs interfere with my work… So, I simply offer the clients what they want (Bulawayo Provider, IDI05, Phase 5)

Such providers reported that their work providing contraceptive methods superseded their personal and socialised beliefs about contraceptive use for young people.

## Discussion

The findings in this study demonstrate patterns in family planning decision-making pathways for young women which may explain the low uptake of long-acting contraceptives among sexually active unmarried young women. Socially constructed and acceptable identities of womanhood contributed to how young women viewed themselves as being appropriate candidates to use contraceptive methods, and access family planning services. The side-effects of hormonal contraceptives, especially LARCs, were framed as a direct threat to attaining ideal womanhood, and LARCs only became a safe and suitable option once fertility had been proven but even then, for some, it remained risky. Many of these contraception decisions were shaped through information and knowledge from influential and trusted sources within the community.

As shown in other research, young women in this study preferred short or medium acting methods, compared to LARCs, when they presented for family planning services, and this reflected in the higher acceptability of shorter acting contraceptives (Willan et al. 2020; Radovich et al. 2018). However, investments to improve access to LARCs have been lauded as being critical to improving unintended pregnancy outcomes among young people (Health Communication Capacity Collaborative 2014). Yet our findings showed that young women considered social acceptability factors that preserved and proved fertility, and not LARC uptake or use.

The priorities regarding young women’s sexual and reproductive concerns were not necessarily the same as those of public health interventions. For public health, the rationale that family planning methods should be a consideration when one is sexually active, regardless of marital status or parity presumes an appeal for hormonal contraceptives, especially LARCS for young women. In contrast, the rationale described by young women and some providers in this study positions LARCs as unsuitable and even threatening to their social priorities. The divergence may explain why, even when family planning methods and services are available to young nulliparous women, their uptake may be low and uneven.

Our findings illuminate the need to focus on how intended beneficiaries’ motivations to access services shape contraceptive uptake, alongside availability. For our study, decision-making was influenced by whether young women perceived themselves to be candidates for hormonal contraceptives, or not. Candidacy here refers to the ways in which individuals deem themselves to be eligible for accessing and utilising health services (Dixon-Woods et al. 2006). In our study, a few unmarried and sexually active young women’s self-determined candidacy aligned with public health rationale-to prevent unwanted pregnancies. Most young women in the study assessed their ‘candidacy’ for family planning by examining social constructions for family planning decision-making and help-seeking. For example, reticence about the effects of LARCs persisted even after fertility had been proven (post-partum) illustrating that while the social threshold for hormonal contraception was the overall demonstration of fertility, the threshold for LARCs may be even higher.

Improving access to and knowledge of family planning services, although helpful, is unlikely to be adequate for young women such as those who participated in this study, even once they know such services are available (Haider et al. 2013). Instead, there is a need to adjust the community-based distribution of contraceptives to suit the context (Marston et al. 2020; Nyundo et al. 2021), reinforcing the call for research and programmes to respond to and accommodate the role of contextual socio-cultural factors in contraceptive behaviours (Agha et al. 2021; Senderowicz 2020; Williamson et al. 2009). Part of such contextualisation will require directly engaging with how to align the health service so that it can directly appeal to, and adjust for, a young person’s candidacy for it.

A key potential adjustment is contextual engagement with the social networks which influence young people’s family planning decisions. The local and social contexts young women engage with before and during contact with a public health intervention (Mackenzie et al. 2013) influence their ‘choices’. Proactively engaging with influential community members, social networks, as well as health providers to address miscon-ceptions within the community and equip young women to become sources of accurate information about hormonal contraceptive use is needed. Addressing the misconception that LARCs threaten fertility may be critical to shifting the prevailing local norms away from undermining the protective opportunities for their use by young women. Unless, and until, public health interventions intentionally engage with social influences and forces, then family planning outcomes for young women may remain compromised.

### Strengths and limitations

Like all research, this study had its limitations. Significantly, we recruited only young women who were already accessing family planning services; and we did not sample young men. Although we were not able to include young women who were not accessing CHIEDZA, participants reflected on what had previously impeded them from taking up family planning options. Many of them were able to explain their continued preference for short or medium-term contraception. Research into young men’s perceptions and beliefs about fertility would enhance understanding of gendered reproductive decision-making.

A strength of the study however was the use of qualitative methods using open and responsive topic guides as well as unstructured formats to generate hypotheses and to develop broader understandings. Another strength was the use of multiple and iterative rounds of data collection which enabled a richer and more detailed understanding of findings.

## Conclusion

Understanding how locally specific social meanings around fertility, and perceived threats, shape both a desirable reproductive sequence and the appeal and perceived ‘suitability’ of different types of contraception for young women is critical. This understanding would enable public health interventions to more effectively engage with, and potentially influence, social norms and structures so as to optimise the acceptability of young women accessing and taking up family planning care, and improve subsequent outcomes amongst young women.

## Figures and Tables

**Table 1:** Qualitative data collection timelines, participants, and methods.

Phase	Sampling strategy	Type of interview participants	Data collection method
1. April 2020	Purposive sample: each province and type of health provider represented	16 health providers (10 women; 6 men)	Phone interviews
2. May–June 2020	Purposive sample: all female youth mobilisers for cluster representation	13 young women	2 Phone interviews 11 In-person interviews
3. July–August 2020	Purposive sample: each province and each type of health provider represented	15 health providers (10 women; 5 men) 8 new; 7 repeat interviews	5 Phone interviews 10 In-person interviews
4. March–May 2021	Purposive sample: for maximum variation by contraceptive type used (short-acting and long-acting)	15 young women clients All in-person interviews	8 Narrative-style in-person interviews 7 topic guide in-person interviews
5. October–November 2021	Purposive sample: each province and type of health provider represented	11 health providers (6 women; 5 men)	In-person interviews

**Table 2:** Key characteristics of the young women interviewed.

	Phase 2 (*n* = 13)	Phase 4 (*n* = 15)
Age	4 aged 16–19 years 9 aged 20–24years Median age (range): 22 (16–24 years)	All 20–25 years Median age (range): 23 (20–25)
Marital status	3 married 5 in a relationship 5 single	12 married 1 divorced 2 in a relationship
Contraceptive use	3 combined oral contraceptives 1 Depo-Provera Injectable 3 Condoms only 6 no contraception	3 combined oral contraceptive 5 Depo-Provera injectable 7 Implants
Parity	5 have children 8 do not have children	All 15 had children
